# Supplementary material for: Piezoelectricity of Bi2Se3 Nanosheet
Source: Nanomaterials (Basel). 2023 Sep 5;13(18):2504. doi: 10.3390/nano13182504 (PMC10535138; doi:10.3390/nano13182504)
Supplement: Supplementary file 1 [file nanomaterials-13-02504-s001.zip › nanomaterials-2577611-supplementary.pdf]

Supplementary data:

Piezoelectricity of Bi_2Se_3 Nanosheet

Tingting Jia [1,2], Liu Yang [2], Juncheng Zhang [3], Hideo Kimura [4], Hongyang Zhao [5], Quansheng Guo [1,*] and Zhenxiang Cheng [6,*]

1. School of Materials Science and Engineering, Hubei University, Wuhan 430062, China
2. Shenzhen Institutes of Advanced Technology, Chinese Academy of Sciences, Shenzhen 518055, China
3. Optics and Optoelectronics Laboratory, Department of Physics, Ocean University of China, Qingdao 266100, China
4. School of Environmental and Material Engineering, Yantai University, Yantai 264005, China
5. Hubei Key Laboratory of Plasma Chemistry and Advanced Materials, Department of Materials Science and Engineering, Wuhan Institute of Technology, Wuhan 430205, China
6. Institute for Superconducting & Electronic Materials, University of Wollongong, Innovation Campus, Wollongong, NSW 2500, Australia

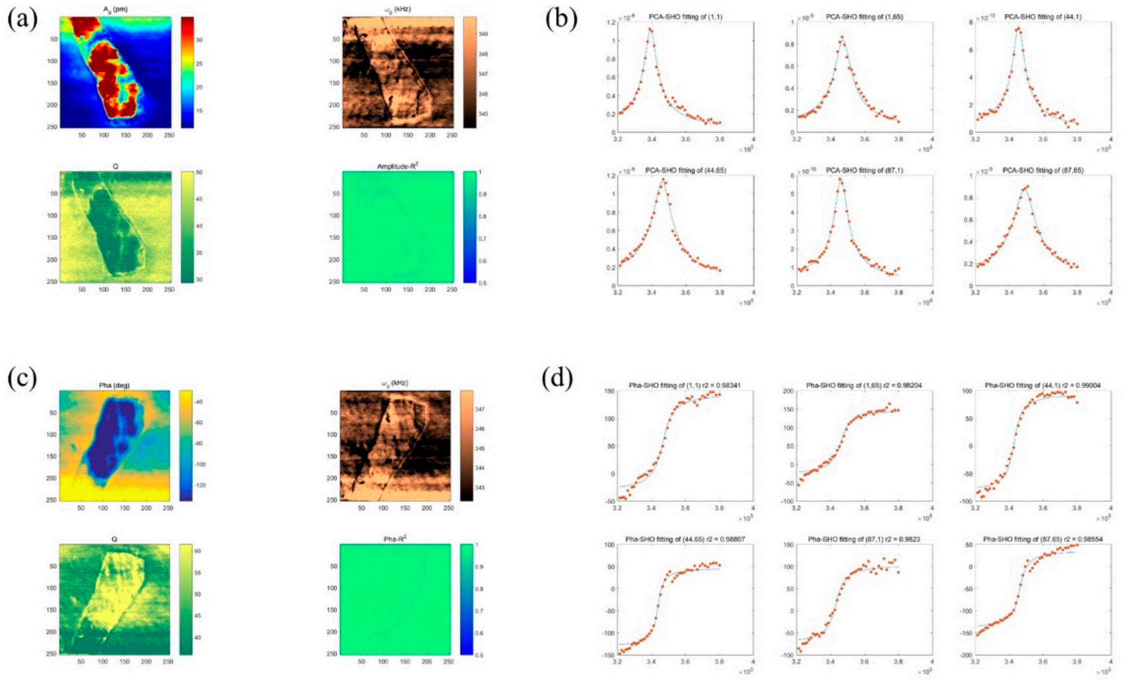

Figure S1. SE-PFM information:(a) Amplitude mapping of A_0, ω_0, Q, R^2. (b) PCA SHO fitting by random. (c) Phase mapping of phase, ω_0, Q, R^2. (b) PCA SHO fitting by random.

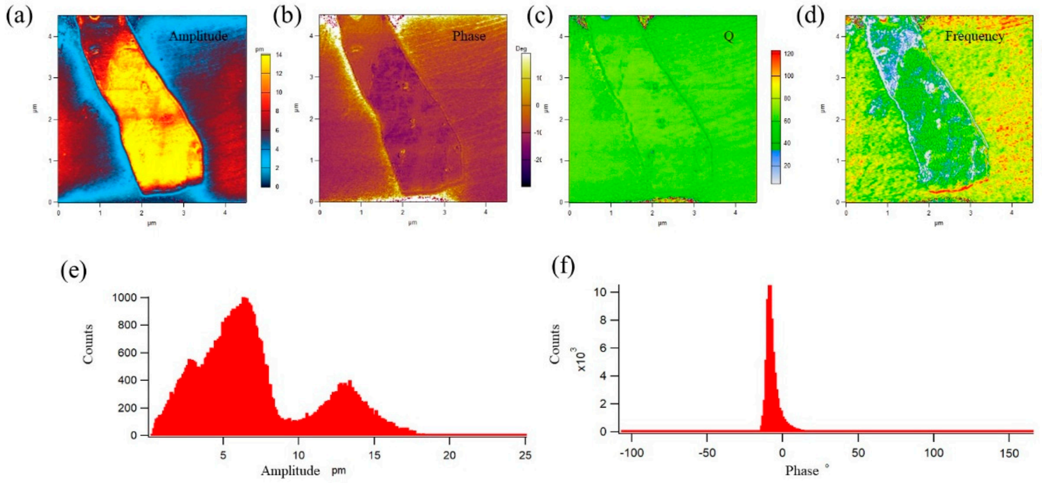

Figure S2. DART in vertical mode details: Mapping of (a) amplitude, (b) phase, (c) Q, (d) frequency. Histogram distributions of (e) amplitude, (f) phase.

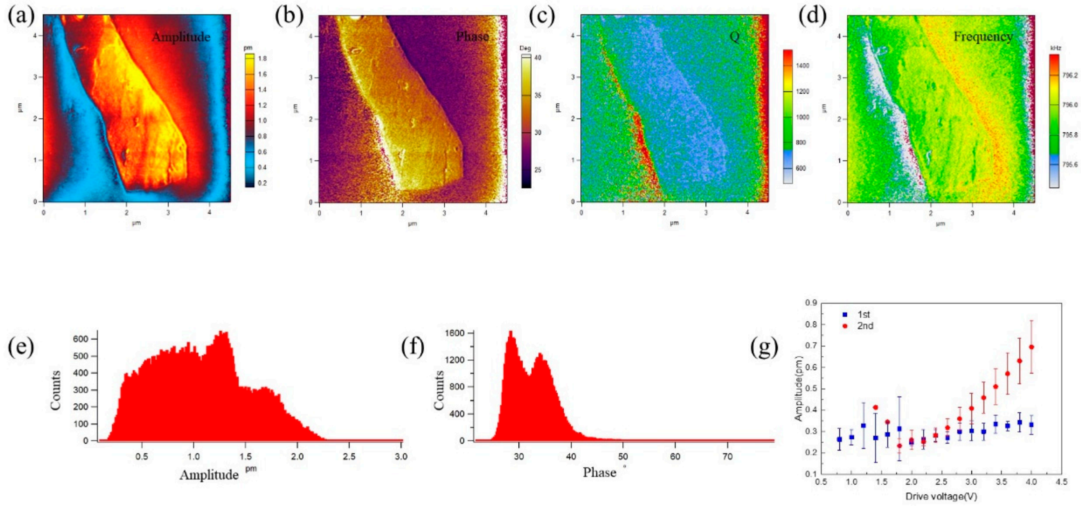

Figure S3. DART in lateral mode details: Mapping of (a) amplitude, (b) phase, (c) Q, (d) frequency. Histogram distributions of (e) amplitude, (f) phase. (g) First and second harmonic responses for lateral PFM.
